# Diagnosing Neurally Mediated Syncope Using Classification Techniques

**DOI:** 10.3390/jcm10215016

**Published:** 2021-10-28

**Authors:** Shahadat Hussain, Zahid Raza, T V Vijay Kumar, Nandu Goswami

**Affiliations:** 1 School of Computer and Systems Sciences, Jawaharlal Nehru University, New Delhi 110067, India; shahad96_scs@jnu.ac.in (S.H.); tvvk@mail.jnu.ac.in (T.V.V.K.); 2 Otto Loewi Research Center for Vascular Biology, Immunology and Inflammation, Medical University of Graz, 8036 Graz, Austria; nandu.goswami@medunigraz.at; 3 Department of Health Sciences, Alma Mater Europea Maribor, 2000 Maribor, Slovenia

**Keywords:** neuro mediated syncope, classification, machine learning, head-up tilt (HUT) test

## Abstract

Syncope is a medical condition resulting in the spontaneous transient loss of consciousness and postural tone with spontaneous recovery. The diagnosis of syncope is a challenging task, as similar types of symptoms are observed in seizures, vertigo, stroke, coma, etc. The advent of Healthcare 4.0, which facilitates the usage of artificial intelligence and big data, has been widely used for diagnosing various diseases based on past historical data. In this paper, classification-based machine learning is used to diagnose syncope based on data collected through a head-up tilt test carried out in a purely clinical setting. This work is concerned with the use of classification techniques for diagnosing neurally mediated syncope triggered by a number of neurocardiogenic or cardiac-related factors. Experimental results show the effectiveness of using classification-based machine learning techniques for an early diagnosis and proactive treatment of neurally mediated syncope.

## 1. Introduction

Syncope is a medical condition resulting in the transient loss of consciousness (LOC) or postural tone with spontaneous recovery. Characterized by certain precipitating factors, warning signs and specific manifestations during the unconscious episode, syncope is a common leading complaint encountered in the emergency department of hospitals [1,2,3]. The most fundamental to the occurrence of syncopal episodes is the short-lived interruption of oxygen supply to the brain that happens primarily due to the transient cessation of blood flow, which is always triggered by the momentarily reversible drop in systemic arterial blood pressure to a level below that needed to sustain cerebral perfusion [4,5]. 

Syncope is mainly classified into three important categories, viz. reflex, cardiovascular and orthostatic hypotension. These groups are further classified into various subgroups depending on the underlying conditions leading to cerebral hypoperfusion. Though numerous possible situations leading to syncope are highlighted in the literature, some cases still exist where even after a thorough assessment it was not possible to assign a single cause of fainting. Reflex or neurally mediated syncope is the most common form of syncope found across all age groups [6]. Usually benign in nature, this syncope is not life-threatening; however, leaving it untreated can be a threat to the quality of life. Orthostatic hypotension (OH) and cardiovascular forms of syncope, on the other hand, are more prevalent among older age groups. In most cases, the severity of OH syncope is mild to moderate. However, the severity of cardiovascular syncope may be life-threatening in nature and requires serious medical attention [7].

The work reported in this paper is restricted to the study and classifications of neurally mediated syncope, where the transient LOC or fainting occurs as a result of an inadequate cerebral blood flow. The defining characteristic of this syncope is the fall in systemic blood pressure (BP) that leads to the reduction in the global cerebral blood flow and, consequently, global cerebral hypoperfusion. The systemic blood pressure is evaluated as the product of the cardiac output (CO) and the total peripheral resistance (TPR). A significant decline in any of the two has the potential to create cessation in cerebral blood flow. However, it has been found that during the induction of syncope, both mechanisms work together in a varying fashion [4]. 

The diagnosis of syncope in itself is a challenging task. This is due to the fact that various other states of altered consciousness such as seizures, vertigo, stroke, coma, etc., also pose the same symptoms as syncope [7]. Additionally, in the diagnostics process, it is crucial to exclude cases of syncope caused by an underlying cardiac disease, as subjects having manifestations of such syncope are at a high risk of severe cardiac-related abnormalities [8]. Thus, evaluating patients with LOC or near LOC and establishing a true syncope is a crucial step in the diagnostic process. The use of high-end computing solutions at this crucial stage of diagnosis is anticipated to add great benefits for resource-constrained healthcare organizations [9]. The advent of Healthcare 4.0 by the usage of artificial intelligence and big data is facilitating a refined diagnostic and treatment procedure [10]. It enables a vast amount of data to be captured and put to work in applications facilitated by machine learning models and, thus, provides a significant gain in the cost and efficiency of healthcare services [11].

The objective of this paper is to diagnose neurally mediated syncope using real-life physiological data collected through the head-up tilt (HUT) test [2]. Classification-based machine learning techniques have been widely and successfully used for diagnoses based on healthcare data [12,13,14,15]. In this regard, a syncope classification model is proposed for classifying syncope and non-syncope events. This work draws its origin from [16], where it was established that syncope classification can be performed using a Support Vector Machine (SVM)-based machine learning algorithm. However, there are many other machine learning algorithms that need to be evaluated for the purpose of syncope classification for an effective and efficient syncope detection. This work uses the dataset used in [16] to present an exhaustive exploration and comparison of some very relevant machine learning classification techniques, including the SVM, for syncope classification. The aim of the study is to further analyze the performance of the SVM with other machine learning peers in this context to explore their suitability in syncope classification.

The remaining paper is organized as follows: Section 2 presents the syncope classification model. Section 3 presents the experimental results, followed by a discussion in Section 4. Section 5 presents the conclusion and future directions.

## 2. Syncope Classification Model 

In this paper, classification techniques, viz. the Decision Tree (DT), Gaussian Naïve Bayes (GNB), k-Nearest Neighbor (k-NN), Multinomial Naïve Bayes (MNB), Support Vector Machine (SVM) and Logistic Regression (LR) were applied to patients’ physiological data. The data utilized for this research were collected from a total of 687 patients, who underwent a HUT test in a purely clinical setting at the Medical University of Graz, Auenbruggerplatz-2, A-8036 Graz. The raw data recorded in the test were preprocessed using statistical methods. The above-mentioned classification algorithms were applied to the preprocessed data. Thereafter, comparisons of these classification algorithms were carried out on five performance measures, viz. the accuracy, precision, recall, F1-score and AUC-ROC curve. 

**Figure 1:**
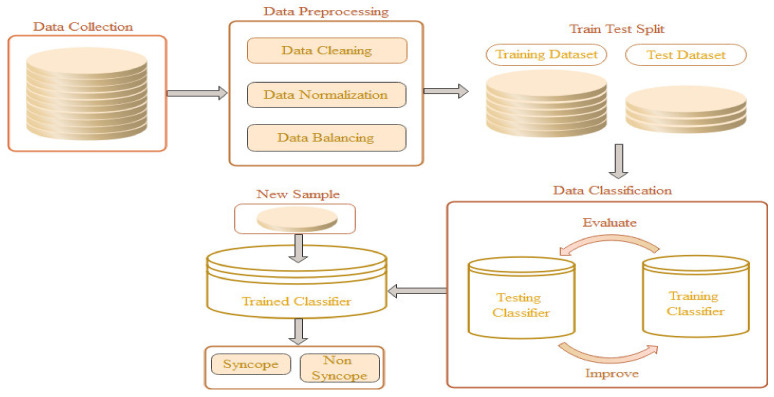
Flow diagram of SCM.

The proposed model, referred to as the syncope classification model (SCM), primarily depends on two central hypotheses. First, the etiology of syncope can be derived by continuous electrocardiographic signals along with beat-to-beat statistics of blood pressure. Second, mathematical modeling and machine learning algorithms can provide a near-accurate diagnosis of autonomic dysfunctional syncope. A flow diagram representing the working of the model is shown in Figure 1. 

### 2.1. Data Collection

The data utilized for this research were obtained from patients who routinely underwent tilt table testing at the Syncope Clinic, General Hospital (LKH), Knittelfeld, Austria. This is a referral center for the assessment of syncopal episodes. The data were collected from a total of 687 patients in a purely clinical setting. All these patients had histories of syncope or dizziness upon standing up. Accordingly, patients having a history of at least a single episode of syncope were included in the study. Informed written consent was obtained from all subjects involved in the study.

The patients, on arrival to the hospital, were instrumented with blood pressure and electrocardiographic sensors. Specifically, hemodynamic responses, such as the heart rate (HR) and mean arterial pressure responses at baseline and at the development of orthostatic intolerance during tilt table testing, were measured. The inclusion and exclusion criteria for patients undergoing tilt table testing were strictly followed. Further, in this exploratory study, continuous and non-invasive beat to beat HR and BP measurements were recorded. Data recorded through sensors were saved digitally with the help of analog to digital converters called the Task Force Monitor (CNSystems, Graz, Austria).

#### 2.1.1. HUT Test

A HUT test is a stimulating test induced to evaluate a patients’ susceptibility to neurally mediated or vasovagal syncope. The test is simply based on an orthostatic stimulus that causes blood to be drained down in lower extremities and, subsequently, vasovagal syncope being ensued to the susceptible individuals. It has been established that the systemic BP of 50–60 mmHg at the heart level or 30–45 mmHg at the brain level in the upright position for 30–45 min can potentially trigger the cessation of cerebral blood flow and, thus, loss of consciousness (LOC) [4,6]. Central hypovolemia, because of blood pooling in lower extremities, is believed to be the triggering mechanism behind the influx of this syncope. The mechanism has extensively been covered in literature by [17,18]. 

Numerous methodologies are being adopted for HUT table testing. Primarily, it is performed in two stages: first, as a drug-free HUT of an elongated duration, followed by a provocative pharmaceutical agent-administered HUT of a shorter duration. A tilt table is, basically, a flat top bedding surface containing footplates and safety straps mounted over it. The tables are fitted with a manual or automatic tilting mechanism that supports calibrated tilt angles of 60° to 80° in a quick span of time. Initially, patients were directed to lie supine on the table before it was started to be tilted upward. The rationale behind the whole action is that a sudden change in posture sometimes induces the neurally mediated syncope that is characterized by a sudden drop in heart rate and blood pressure. The tests are usually supervised by a physician or technician having expertise in the management of the test and its potential complications. A standard tilt test protocol was used for this study. Following a baseline of 20 min, the participants were tilted for 20 min. If no syncopal signs or symptoms developed after 20 min, the tilt test was negative. However, if the signs or symptoms of syncope developed at any time, the tilt table was immediately returned back to the supine position and the tilt test was classified as positive. No pharmacological interventions were provided in this study. The test ended after the induction of presyncope or syncope related to intolerable hypotension [19,20,21]. The test for this study found three main underlying mechanisms responsible for the triggering of the induction of syncope, viz. a sudden drop in blood pressure, drop in heart rate resulting in a drop in blood pressure and a continual drop in blood pressure as reported in [16]. 

#### 2.1.2. Physiological Indicators

The HUT test concluded with the findings that, out of 687 patients, only 96 patients were recognized to have the induction of syncope, while the remaining 592 patients were able to keep control of their BP and HR. A total of 48 different physiological indicators against each individual were recorded in a proper format as reported in [16]. These indicators were majorly grouped under five subgroups, viz. BeatStats, Cardiac BeatStats, HRV Stats, dBPV Stats and sBPV Stats. The subgroups, BeatStats and Cardiac BeatStat, respectively, were a collection of 11 and 13 indicators, whereas each of the HRV Stats, dBPV Stats and sBPV Stats contained 8 indicators. 

A complete description of all physiological indicators was beyond the scope of this research. However, a brief introduction of each indicator along with their assessment was presented in Appendix A for putting things in perspective.

### 2.2. Data Preprocessing

The physiological dataset, discussed above, had missing values for indicators for some patients. The missing values were replaced by the average value of the respective indicator of all patients. Further, since the values for different indicators were in different scales for the patient instances, the values for each of the indicators were normalized using the min–max normalization technique to lie between 0 and 1. Furthermore, out of the total of 687 patient instances, 96 instances belonged to the syncope class and 591 instances belonged to the non-syncope class. Thus, the dataset exhibited a class imbalance, which in turn could lead to poor performance of the classification model. There exist several oversampling techniques to address this class imbalance problem, and one amongst them, the Synthetic Minority Oversampling Technique (SMOTE) [22], was used to address the class imbalance problem in the physiological dataset used in the SCM. SMOTE over-sampled the minority class by creating a synthetic example of an instance instead of replacing it. It randomly selected a sample instance in the minority class and computed its k-nearest neighbors. It, then, generated a line segment between the selected instance and computed the k-nearest neighbors and chose synthetic instances falling within these lines and added them to the dataset. This process continued until the required additional samples were added to the dataset. The resultant dataset hereafter in this paper was referred to as the syncope dataset.

### 2.3. Data Classification

Six supervised machine learning algorithms, viz. Decision Tree, Multinomial Naïve Bayes, Gaussian Naïve Bayes, k-Nearest Neighbor, Support Vector Machine and Logistic Regression, were applied over the normalized balanced physiological syncope dataset in order to classify it into syncope events or non-syncope events. The aforementioned classification algorithms were briefly discussed in Appendix B.

The K-fold cross-validation technique was used for training and testing the employed classification models. In this validation technique, the input dataset was divided into k subsets or folds of disjoint sets. Classification models were trained with data in k-1 folds and an evaluation was carried out using the data in the remaining one-fold. The whole process iteratively ran k times gave each fold at least one and at most one chance to be used for testing. In this process, the syncope dataset was divided into ten disjoint sets of data, shown in the form of dotted and crisscross bars. Dotted bars represent the subsets of data that were iteratively employed for training the model, while the crisscross bar represents the dataset that was used for testing it. The process ran *ten* times for different sets of training and testing data.

### 2.4. Performance Metrics

The performance of the classification models was based on the elements of matrix imported from information retrieval, i.e., confusion matrix, which was a 2 × 2 matrix of four elements, viz. True Positive (TP), False Positive (FP), False Negative (FN) and True Negative (TN), as shown in Table 1. It was used for the binary classification problem, where positive and negative implied a syncope event and non-syncope event, respectively. 

The algorithms used for the syncope classification models in this work were compared based on five performance measures, viz. accuracy, precision, recall, F1-score and AUC-ROC [23,24], which were computed using the elements of the above-mentioned confusion matrix. These were standard performance measures used to evaluate machine learning models, which were briefly discussed in Appendix C.

## 3. Experimental Results

Experiments were carried out to ascertain the benefits of using machine learning models and compare them on various performance parameters. The hardware, software and API specifications used in the experimental set-up were listed in Appendix D. Further, the model-related parameter values considered for experimentation were presented in Appendix E.

The syncope dataset, as discussed in Section 3, was used for experimentation. Since these data had many missing values, they were normalized using min–max normalization as discussed in Section 2.2. Further, in order to balance the dataset, the SMOTE oversampling technique, as discussed in Section 2.2, was used. Next, stratified 10-fold cross-validation was used for training and testing the syncope dataset using the classification-based machine learning models discussed in Appendix B. For each of these machine learning models, various performance matrices, such as the accuracy, precision, recall, F1-Score and AUC-ROC curve as discussed in Appendix C, were computed and are given in Table 2, Table 3, Table 4, Table 5 and Table 6, respectively. To provide a comprehensive view, minimal values (Min), maximal values (Max), mean values (Mean) and standard deviation (SD) were computed, as given in these five Tables, while considering each fold for testing at least and at most once across 10 executions. 

In the case of the mean accuracy, LR, the SVM and DT performed comparatively better than the other models, with LR performing the best amongst them. However, the mean accuracy of MNB was significantly low. Further, it can be noted that LR, the SVM and DT had a lower SD value and achieved the maximum accuracy value of 1. Furthermore, the minimum accuracy value achieved by these models was also comparatively higher than other models. Thus, it can be inferred that if accuracy was the key parameter, then LR, the SVM and DT could be used for syncope classification.

In the case of the mean precision, k-NN, DT and LR performed comparatively better than the other models, with k-NN performing the best amongst them. However, the mean precision of MNB was significantly low. Further, it can be noted that though the maximum precision value achieved by all the models was one, they varied in their minimum precision value and SD value with DT, k-NN and LR having a comparatively lower SD value. Thus, it can be inferred that if precision was the key parameter, then DT, k-NN and LR could be used for syncope classification.

In the case of the mean recall, MNB, LR and GNB performed comparatively better than the other models, with MNB performing the best amongst them. However, the mean recall of k-NN was significantly low. Further, it can be noted that the maximum recall value achieved by all the models, except k-NN, was one. However, the minimum and maximum recall value of k-NN was significantly low and had the maximum SD amongst all the models. Furthermore, MNB, LR and GNB had a comparatively lower SD value. Thus, it can be inferred that if recall was the key parameter, then MNB, LR and GNB could be used for syncope classification.

In the case of the mean F1-score, LR, the SVM and DT performed comparatively better than the other models, with LR performing the best amongst them. Further, it can be noted that the maximum F1-score value achieved by these models was 1. However, the Min, Max and Mean F1-score value of MNB and k-NN was significantly low. Furthermore, LR, the SVM and DT had a comparatively lower SD value. Thus, it can be inferred that if F1-score was the key performance parameter, then LR, the SVM and DT could be used for syncope classification. 

While comparing the machine learning on the above-mentioned performance parameters, it was observed that LR performed comparatively better in terms of accuracy, precision and F1-score and was comparatively as good as the best performing model k-NN in terms of recall. Further, in order to ascertain the performance of LR on different thresholds, in comparison to other models, area under the ROC curve (AUC-ROC) value was computed and is given in Table 6.

In case of the mean AUC-ROC value, LR performed comparatively better than the other models. Further, it can be noted that though the maximum AUC-ROC value achieved by LR, DT and the SVM was one, the mean AUC-ROC value and SD value of LR was comparatively better than DT and the SVM. Thus, it can be inferred that the overall performance of LR was comparatively better than the other models and, thus, could be used effectively for syncope classifications.

## 4. Discussion

Classifying syncope and non-syncope events, based on true physiological data, have rarely been dealt with by researchers. However, with the recent advent of huge computing capabilities, data-based analytics and the diagnosis of cardiac-related abnormalities have become a major domain of research across healthcare organizations. 

A model for vasovagal syncope classification using the Support Vector Machine (SVM)-based classification was presented in [16]. However, the work was limited to the use of the SVM in classification and lacked the exploration of other supervised machine learning models which could result in a better classification. The research work [25] focused on the multi-class classification and clustering of syncope based on the heart rate (HR) and the blood pressure (BP) data collected using the HUT test. The classifications were performed by random forest classifier, whereas the K-medoid [26] technique was used for clustering purposes. The result shown in the work was promising and facilitated a clear viewpoint of the autonomic system, while analyzing pathophysiological indicators. However, the result presented in the work was derived by a single classifier and was based on time series measurements of only HR and BP data. Some common forms of syncope were classified for diagnosis and treatment by [27,28,29]. Since these classifications were derived from the laboratory findings and observation of an individual physician, they were phenomenological and lacked consistency in terminology.

A work, based on the random forest algorithm for the differentiation between syncope and other common causes of transient LOC, was presented by [30]. However, its results were not based on the physiological indicators; instead, they were based on the dataset generated by the outcomes of response to detailed questionnaires about transient LOC. 

Another work aiming for the early prediction of syncope during the HUT test was reported by [31]. This work predicted the outcome of the HUT test based on data generated in the first 15 min of the test. However, the results were exclusively based on the dynamic interaction between the RR interval and the amplitude of systolic blood pressure.

A Natural Language Processing algorithm to identify syncope from the emergency department (ED) electronic medical records (EMRs) was reported in [32]. Here, the models claimed an impressive outcome towards the automatic identification of syncope for large populations, providing a 96% reduction in analysis time as compared to the manual reviews of EMRs. However, the results were based only on the EMRs of the patients visiting the emergency department.

The work carried out in this paper offered a near accurate mechanism for the diagnosis of neurally mediated syncope based on patient’s data collected through a full-scale HUT test in a purely clinical setting, as mentioned in Section 2.1. This work focused on the application of widely used classification-based machine learning algorithms, DT, GNB, k-NN, MNB, the SVM and LR, on a syncope dataset comprising 48 physiological indicators.

Table 7 summarizes the performance of classifiers for each performance metric based on the results presented in Section 3. The columns Max (min), Max (max), Max (mean) and Min (SD) denote the name of the classifiers, amongst all classifiers, which best evaluated the results against each of the performance metrics.

It can be inferred from Table 7 that LR performed comparatively better in terms of accuracy, recall, F1-score and AUC-ROC, and performed reasonably well in terms of precision. Further, the performance of LR across multiple thresholds, computed using AUC-ROC, was comparatively better than the other models. Thus, it can be stated that the overall performance of LR was the best and it could be used for the diagnosis of neurally mediated syncope. 

This work can be improved by ascertaining the indicators and their combinations that are relevant for classifying neurally mediated syncope. Different medical conditions [33], including polypharmacy, should be considered for future studies, particularly associated with older persons [34,35,36,37], as there are dissimilar cardiovascular patterns in healthy participants [38]. Further, the generalizability of data considering the effect of sex [39,40,41], seasons [42] or across races on reproducibility can be assessed while considering the inter-individual differences in hemodynamic responses and time to collapse [43]. Furthermore, a detailed analysis of HR and BP features could be performed to evaluate whether they can have a predictive value on their own or not.

## 5. Conclusions

The work carried out in this paper emphasized the benefits of using classification-based machine learning models for the diagnosis of neurally mediated syncope. Amongst all the classification models, the LR-based classification model performed the best and could appropriately be used for classifying neurally mediated syncope.

## Figures and Tables

**Table 1:** Confusion matrix.

	Actual Value		
**Predicted Value**		Positive	Negative
	Positive	TP	FP
	Negative	FN	TN

The four elements of the matrix are described as: true positive (TP): the predicted syncope event classified correctly. True negative (TN): the predicted non-syncope event classified correctly. False positive (FP): the predicted syncope event classified incorrectly. This is also called a type 1 error. False negative (FN): the predicted non-syncope event classified incorrectly. This is also called a type 2 error.

**Table 2:** Accuracy.

Classifiers	Min	Max	Mean	SD
Decision Tree	0.956521	1.00	0.978197	0.012331
Gaussian Naïve Bayes	0.927536	0.985507	0.959292	0.019032
k-Nearest Neighbors	0.855073	0.970588	0.914258	0.036931
Multinomial Naïve Bayes	0.397059	0.985507	0.575234	0.156069
Support Vector Machine	0.955882	1.00	0.975256	0.013813
Logistic Regression	0.970588	1.00	0.989812	0.013814

**Table 3:** Precision.

Classifiers	Min	Max	Mean	SD
Decision Tree	0.833333	1.00	0.953081	0.065427
Gaussian Naïve Bayes	0.60	1.00	0.792345	0.114308
k-Nearest Neighbors	0.875	1.00	0.959230	0.054528
Multinomial Naïve Bayes	0.333333	1.00	0.525029	0.183290
Support Vector Machine	0.75	1.00	0.899626	0.087672
Logistic Regression	0.75	1.00	0.922723	0.094551

**Table 4:** Recall.

Classifiers	Min	Max	Mean	SD
Decision Tree	0.60	1.00	0.866866	0.130834
Gaussian Naïve Bayes	0.80	1.00	0.939061	0.064175
k-Nearest Neighbors	0.20	0.70	0.431945	0.174117
Multinomial Naïve Bayes	0.80	1.00	0.977658	0.062855
Support Vector Machine	0.80	1.00	0.909733	0.077714
Logistic Regression	0.80	1.00	0.976079	0.063083

**Table 5:** F1-score.

Classifiers	Min	Max	Mean	SD
Decision Tree	0.75	1.00	0.900775	0.076146
Gaussian Naïve Bayes	0.727273	0.96	0.858069	0.072367
k-Nearest Neighbors	0.333333	0.823529	0.574271	0.176757
Multinomial Naïve Bayes	0.235294	0.888889	0.434743	0.175213
Support Vector Machine	0.80	1.00	0.902410	0.062612
Logistic Regression	0.80	1.00	0.949312	0.070388

**Table 6:** AUC-ROC.

Classifiers	Min	Max	Mean	SD
Decision Tree	0.80	1.00	0.928496	0.062632
Gaussian Naïve Bayes	0.884375	0.982142	0.948923	0.028553
k-Nearest Neighbors	0.60	0.85	0.712434	0.086574
Multinomial Naïve Bayes	0.627119	0.90	0.710141	0.076134
Support Vector Machine	0.891379	1.00	0.949001	0.038459
Logistic Regression	0.892188	1.00	0.983263	0.032924

**Table 7:** Classifiers that evaluate desirable outputs across the K-folds.

Measures	Max (Min)	Max (Max)	Max (Mean)	Min (SD)
Accuracy	LR	DT, SVM, LR	LR	DT
Precision	k-NN	DT, GNB, k-NN, MNB, SVM, LR	k-NN	k-NN
Recall	k-NN	DT, GNB, MNB, SVM, LR	MNB, LR	MNB
F1-score	SVM, LR	DT, SVM, LR	LR	SVM
AUC-ROC	SVM, LR	DT, SVM, LR	LR	GNB

## Data Availability

The data in this study are readily available upon reasonable request to the corresponding author.

## Appendix A

A summary of physiological indicators along with their measuring units are presented in Table A1 [16].

**Table A1:** Physiological indicators of subjects collected in HUT test.

BeatStats		
Acronym	Definition	Units
HR	Heart Rate	Beats/min
SV	Stroke Volume	Liter/beat
CO	Cardiac Output	Liter/min
CI	Cardiac Index	Liter/min/m^2^
SI	Stroke Index	Ml/beat/m^2^
RRI	RR Interval	Seconds
TPR	Total Peripheral Resistance	Pa·sec/m^3^
TPRI	Total Peripheral Resistance Index	Pa·sec/m^5^
dBP	Diastolic Blood Pressure	mmHg
mBp	Mean Blood Pressure	mmHg
sBP	Systolic Blood Pressure	mmHg
Cardiac BeatStats		
ACI	Acceleration Index	m/s^2^
CI	Cardiac Index	Liter/min/m^2^
EDI	End-Diastolic Index	
HR	Heart Rate	Beats/min
IC	Index of Contractility	Seconds
LVET	Left Ventricular Primitive Ejection Time	Milliseconds
LVWI	Left Ventricular Stroke Work Index	Pa.ml/beat/m^2^
SI	Stroke Index	Ml/beat/m^2^
TFC	Thoracic Fluid Content	Liter
TPRI	Total Peripheral Resistance Index	Pa·sec/m^5^
dBP	Diastolic Blood Pressure	mmHg
mBp	Mean Blood Pressure	mmHg
sBP	Systolic Blood Pressure	mmHg
** HRV Stats **		
HF_RRI	High-Frequency RR Interval	Hz
HFnu_RRI	Normalized High-Frequency RR Interval	
LF_HF	Difference Between Low and High Frequency of RR Interval	Hz
LF_HF_RRI	The ratio of Low and High Frequency of RR Interval	
LF_RRI	Low-Frequency RR Interval	Hz
LFnu_RRI	Normalized Low-Frequency RR Interval	
PSD_RRI	Power Spectral Density of RR Interval	W/Hz
VLF_RRI	Very Low Frequency of RR Interval	Hz
dBPV Stats		
HF_dBP	High-Frequency dBP	Hz
HFnu_dBP	Normalized High-Frequency dBP	
LF_HF	Difference Between Low and High Frequency of dBP	Hz
LF_HF_dBP	Ratio of Low and High Frequency of dBP	
LF_dBP	Low-Frequency dBP	Hz
LFnu_dBP	Normalized Low-Frequency dBP	
PSD_dBP	Power Spectral Density of dBP	W/Hz
VLF_dBP	Very Low Frequency of dBP	Hz
** sBPV Stats **		
HF_sBP	High-Frequency sBP	Hz
HFnu_sBP	Normalized High-Frequency sBP	
LF_HF	Difference Between Low and High Frequency of sBP	Hz
LF_HF_sBP	Ratio of Low and High Frequency of sBP	
LF_sBP	Low-Frequency sBP	Hz
LFnu_sBP	Normalized Low-Frequency sBP	
PSD_sBP	Power Spectral Density of sBP	W/Hz
VLF_sBP	Very Low Frequency of sBP	Hz

The description of physiological indicators along with their measuring units is given below:

*Heart Rate (HR_[bpm]_)*: It is the speed of the heartbeat measured as the number of contractions of the heart per minute.

*Stroke Volume (SV_[l/beat]_)*: It is the amount of blood ejected by the left ventricle in one beat.

*Cardiac Output (CO_[l/min]_)*: It is the volume of blood being pumped by the left or right ventricle of the heart in a unit of time. It is measured as the product of stroke volume and heart rate.

*CO_[l/min]_* = *SV_[l/beat]_ × HR_[bpm]_*

*Cardiac Index (CI_[l/min/m_^2^_]_)*: It is a hemodynamic parameter that relates the cardiac output (CO) from the left ventricle in one minute to body surface area (BSA); thus, relating heart performance to the size of the individual. The unit of measurement is in liters per minute per square meter (L/min/m^2^). Cardiac index is measured as given below:

*CI_[l/min/m_^2^_]_* = *CO_[l/min]_*/*BSA_[m_^2^_]_*

where Body Surface Area *(BSA_[m_^2^_]_)* is the surface area of a human body empirically calculated in terms of height and weight of the body.

*BSA_[m_^2^_]_ = Weight_[kg]_ [x] × Height_[cm]_[y] × [z]*

*x, y* and *z* are empirically derived parameters.

*Stroke Index (SI_[ml/beat/m_^2^_]_)*: It is the normalized stroke volume corresponding to each unit of body surface area.

*SI_[ml/beat/m_^2^_]_* = *SV_[l/beat]_*/*BSA_[m_^2^_]_ × 1000*

*RR Interval (RRI_[s]_)*: It is the time elapsed between two consecutive R waves of the QRS signal on the electrocardiogram. It is a function of the intrinsic properties of the sinus node as well as autonomic influences.

*Total Peripheral Resistance (TPR _[_*_Pa·s·m_^−3^*_]_)*: It is a measure of the total resistance observed in the blood flow produced by the entire vascular system. In general, this equates to the force applied by the heart in order to pump the blood.

*Total Peripheral Resistance Index (TPRI _[Pa·s·m_^−^*^5^*_]_)*: It is the measure of TPR corresponding to each part of the body surface area.

*TPRI _[Pa·s·m_^−5^_]_* = *TPR*_*[*Pa·s·m_^−3^_*]/*_*BSA_[m_^2^_]_*

*Diastolic Blood Pressure (dBP_[mmHg]_)*: It is the pressure in the arteries between two consecutive heartbeats.

*Systolic Blood Pressure (sBP_[mmHg]_)*: It is the pressure in the arteries during the contraction of the heart muscle.

*Mean Blood Pressure (mBP_[mmHg]_)*: It is the average blood pressure in an individual during a single cardiac cycle. It has the same unit as dBP or sBP and is evaluated as:

*mBP_[mmHg_* = (2/3) × *dBP_[mmHg]_* + (1/3) × *sBP_[mmHg]_*

*Acceleration Index (ACI_[ms_^−^*^2^*_]_):* It is the highest acceleration of blood flows in the aorta.

*End-Diastolic Index (EDI_[_*_ml/m_^2^_]_*)*: End-diastolic volume (EDV) is the volume of blood in the right and/or left ventricle just before systole. EDV is often used synonymously with preload that refers to the length of the sarcomeres in cardiac muscle prior to its contraction. The amount of EDV corresponding to each unit of body surface area is defined as an end-diastolic index (EDI). It increases the preload on the heart and, thus, increases the stroke volume. It is evaluated automatically using cardiac magnetic resonance imaging (MRI) software.

*Index of Contractility (IC_[s]_)*: It is the assessment of myocardial contractile forces depending upon the pressure P in the left ventricular and its first derivative dP/dt between the time from onset of contraction to the time t_d_ when it attains dP/dt_max_, on the isovolumic pressure curve.

*Left Ventricular Ejection Time (LVET_[_*_ms*]*_): It is the time duration between the opening and closing of the aortic valve, which in turn is evaluated by the pressure difference across the valve.

*Left Ventricular Stroke Work Index (LVWI)*: It describes the function of the left ventricular in a numeric form. LVWI highlights the increment of a workload on the left ventricle in aortic stenosis.

*LVWI* = *SI_[ml/beat/m_^2^_]_* × *(LVSP_[mmHg]_ − LVEDP_[mmHg]_).*

where, (*LVSP*) and (*LVEDP*) are the left ventricular mean systolic pressure and left ventricular end-diastolic pressure, respectively.

*Thoracic Fluid Content (TFC_[l]_)*: It is a non-invasively assessed parameter by thoracic electrical bio-impedance. TFC indicates the total volume of chest fluid, particularly in the lungs. Pulmonary perfusion can directly be estimated from TFC in the case of any lack of pleural or pericardial effusion.

Table A1 contains subgroups having physiological indicators from heart rate variables (HRV Stats) and blood pressure variables (both dBPV Stats and sBP Stats). The spectral analysis of heart rate and blood pressure on a beat-to-beat basis presents the sensitive assessment of cardiovascular functions that provides clinical diagnostic and prognostics markers [44]. The TFM employed for the measurement of cardiovascular parameters in this work automatically calculated the power spectral analysis of HRV Stats and BPV Stats accordingly. Short recordings of HRV and BPV were primarily observed in three separate frequency bands of oscillations, namely, very low frequency (VLF), low frequency (*LF*) and high frequency (*HF*). The separation considered for each frequency band was 0.01 to 0.20 Hz for VLF, 0.20 to 0.75 Hz for *LF* and from 0.75 to 250 Hz for HF. In short-duration autonomic regulations, VLF was considered to be insignificant, as it hardly played any major role in the final result. Thus, *LF* and *HF* were the only significant measures used to quantify the parasympathetic and sympathetic regulations [45]. Moreover, further studies suggest that for the precise derivation of outcomes from HRV and BPV statistics, the only measures of *LF* and *HF* were not adequate. Therefore, in addition to the absolute value of *LF* and *HF*, their normalized form *LFnu* and *HFnu* were also computed by the TFM.

*LFnu = LF/(HF + LF + VLF) and*

*HFnu = HF/(HF +LF + VLF)*

Since *VLF* was also considered in the quantification of *LFnu and HFnu,* thus,

*LFnu + HFnu < 1.*

The alterations in the spectra by cardiovascular disturbances in the autonomic circulatory regulation affected the proportion of frequency in the total spectrum power. Therefore, the power spectral density (PSD) was also considered as a quantitative indicator for autonomic regulation. Furthermore, the ratio between *LF* and *HF* was also computed by the TFM as a direct proportionality drawn between a normalized value of either of the spectral bands and *LF*/*HF* ratios.

As discussed above, a total of eight indicators, viz. VLF, LF, HF, LF/HF, LF-HF, LFnu, HFnu and PSD, were computed by the TFM for a spectral analysis of HRV and BPV. These indicators were separately computed for each of the three subgroups HRV Stats, sBPV Stats and dBPV Stats.

The HUT test process records data of every individual on 48 physiological indicators discussed above. All these measurements were recorded over a dedicated task force monitor (TFM), which facilitates continuous and reproducible measurements of HR and BP.

## Appendix B

Machine learning models used for classifying syncope and non-syncope are given below:

Decision Tree [46]: It is a tree-structured supervised learning method used both for solving the classification and regression problems. Based on the top-down greedy approach, it breaks the dataset into small subsets and, simultaneously, develops an incremental decision tree. A decision tree (DT) consists of three components, viz. the internal node, leaf nodes and branch, where internal nodes represent the feature, leaf node signifies the outcome and branch dictates the decision rules. The selection of best attributes for root nodes and internal nodes depends on the measures of decrease in impurity or randomness in the dataset. For predicting the class for the input syncope data, the decision tree, for the given syncope dataset, is traversed from the root node to the leaf node based on the condition met by the input data. The input data is classified as a syncope and non-syncope based the label of the leaf node.

Multinomial Naïve Bayes [47]: The multivariate event model of Naïve Bayes, referred to as Multinomial Naive Bayes (MNB), is a probabilistic learning method, based on Bayes Theorem, where the adjective Naïve is used because of the assumption that the features are independent of each other. In case of the syncope dataset, MNB can be used to evaluate the probability of the occurrence of syncope to a patient, s, amongst the class of patients, c, as:

$$P(c│s)\propto \underset{1\leq k\leq n_{s}}{\prod }{P(x}_{k}\left|c\right)$$

here ${P(x}_{k}\left|c\right)$ represents the conditional probability of feature $x_{k}$ occurring in a dataset of the class of patient c. ${P(x}_{k}\left|c\right)$ measures the contribution of feature $x_{k}$ in finding the correct class c. P(c) is the prior probability of the occurrence of syncope in class c. When features show no clear evidence about one class versus another, the class having higher prior probability is chosen. $n_{s}$ represents the number of features considered for classifications. This algorithm performs better when the features take discrete values and the features are independent of each other.

Gaussian Naïve Bayes [48]: The classification model based on the extension of the Naive Bayes model to real-valued attributes using Gaussian distribution is referred to as the Gaussian Naïve Bayes (GNB) model. It assumes that data described by Gaussian distribution have no co-variance between dimensions. This model can be used to compute the mean and standard deviation of all indicators into the syncope dataset and find the Gaussian probability density function of indicators $x_{i}$ for a new patient x as:

$$p\left(x_{i}\right)=\frac{1}{\sigma \times \sqrt{2\pi }}e^{\frac{-1}{2}{\left(\frac{x_{i}-\mu }{\sigma }\right)}^{2}}$$

where μ and σ are the mean and standard deviation, respectively.

The probable class C of patient x is finally computed by the highest value of posterior probability (P) as:

$${P(C}_{k}{|x)=\max(p(x|C}_{k}))$$

where *k* is the number of possible classes and ${p(x|C}_{k})$ is the probability density of patient x corresponding to $C_{k}$.

k-Nearest Neighbor [49]: This is a supervised learning algorithm that classifies data to a class that have data in a close proximity or neighborhood. The distance between the input data point and data points in the dataset are used to decide the k-nearest neighbor (k-NN). In the case of the syncope problem, k-NN can be used to classify patients into the syncope or non-syncope class depending on which of these classes obtains the majority of votes from the data instances comprising the *k*-nearest neighbors.

Support Vector Machine [50]: It is a linear classifier that performs the classification task by creating a hyperplane in a higher dimensional space that, optimally, splits the data into two groups. The support vector machine (SVM) tries to find an optimal hyper plane corresponding to *m* given training samples$\{\left(x_{1}{,y}_{1}\right),\ldots \left(x_{m}{,y}_{m}\right)$}, where $x_{i}$∈ R^N^ and $y_{m}$∈ {−1, 1}, for the linear decision function *f*(*x*) = *w*· *x* + *b*, by evaluating weight *w* and bias *b*. In the case of the syncope dataset, the SVM can be used to classify a patient by choosing the hyperplane having the largest margin, which is evaluated as the closest distance from the data points to the decision boundary.

Logistic Regression [51]: It is a supervised learning technique that considers a logistic function or the sigmoid function to map the predictions to probabilities. Logistic regression (LR) uses a logistic function defined as:

$$f\left(x_{i}\right)=\frac{1}{{1+e}^{{-(x}_{i})}}$$

where $x_{i}$ are indicators in the dataset.

The function, when plotted on a graph, is an S-shaped curve between 0 and 1. For the classification of patients into the syncope or non-syncope class, the input data features are passed through the sigmoid function that computes its probability ranging between 0 and 1. The algorithm works with a threshold value which is used to classify patients based on the output of the probabilistic function given as:

$$\log(\frac{f(x)}{1-f(x)}{)=b}_{0}{+b}_{1}{\;\times \;x}_{1}{+b}_{2}{\;\times \;x}_{2}+\ldots b_{n}{\;\times \;x}_{n}\;+\varepsilon$$

where $b_{0}$ is the intercept on the *y* axis, $b_{1}{,b}_{2},\ldots b_{n}$ are the linear coefficients corresponding to each one of the indicators and *ε* is the random error.

## Appendix C

Performance metrics for comparing syncope classification models are given below:

Accuracy: It is defined as the correct fraction of total predictions computed by the model. For a binary classification problem, accuracy is evaluated as:

$$\mathrm{Accuracy}=\frac{\mathrm{TP}+\mathrm{TN}}{\mathrm{TP}+\mathrm{TN}+\mathrm{FP}+\mathrm{FN}}$$

Recall: It is defined as the ratio of correct positive predictions to the actual positive samples. It is also called the true positive rate (TPR) or sensitivity. The higher value of recall indicates the better performance of the model.

$$\mathrm{Recall}/\mathrm{Sensitivity}/\mathrm{TPR}=\frac{\mathrm{TP}}{\mathrm{TP}+\mathrm{FN}}$$

Precision: It is defined as the ratio of correct positive predictions to the total positive predictions determined by the classifier. The higher the value of precision, the better is the performance of the model.

$$\mathrm{Precision}=\frac{\mathrm{TP}}{\;\mathrm{TP}+\mathrm{FP}}$$

F1–score: It is the harmonic mean of recall and precision. Ranging from zero to one, a higher value of F1-score indicates a better performance. This measure is widely used for the evaluation when models generate a high recall and low precision or vice versa.

$$F1-\mathrm{Score}=\frac{2}{\frac{1}{\mathrm{Recall}\;}+\frac{1}{\mathrm{Precision}}}\;=\frac{2\;\times \;\mathrm{Recall}\;\times \;\mathrm{Precision}}{\mathrm{Recall}+\mathrm{Precision}}$$

False Positive Rate: It is defined as the ratio of incorrect positive predictions to the actual negative samples.

$$\mathrm{FPR}=\frac{\mathrm{FP}}{\mathrm{FP}+\mathrm{TN}}$$

Area Under the ROC Curve (AUC-ROC): A receiver operating characteristics (ROC) curve is a graph that depicts the performance of a classification model for all classification thresholds. The curve is plotted between TPR on the *y*-axis and the FPR on the *x*-axis. The area under the ROC curve (AUC-ROC) is the performance metric for measuring the capability of binary classifiers. A higher AUC-ROC signifies the better performance of the classifier.

## Appendix D

Hardware, software and API specifications used for experimentation are given in Table A2.

**Table A2:** System specifications.

Hardware	Specifications	Software	Specifications
Processor	Core i5	Windows	64-bit Windows 10
Processor Clock Speed	1.8 GHz	Scikit learn	0.20.3
Number of Cores	4	Pandas	0.23.4
RAM	8 GB	NumPy	1.14.3
Cache Memory	6 MB	Matplotlib	3.0.2
Processor Architecture	64 bit	Seaborn	0.11.1
Processor Variant	8265U	imblearn	0.00

## Appendix E

Parameters used by syncope classification model are given in Table A3.

**Table A3:** Parameter values considered by classification models.

**SVM Parameters**							
**Parameter**	**Value**	**Parameter**	**Value**	**Parameter**	**Value**	**Parameter**	**Value**
C	2	kernal	Linear	degree	3	gamma	Auto
coef0	0.0	shrinking	True	probability	False	tol	0.001
cache_ size	200	class_ weight	None	verbose	False	max_iter	−1
decision_ function_ shape	ovr	break_ties	False	random_ state	None		
**LR Parameters**							
**Parameter**	**Value**	**Parameter**	**Value**	**Parameter**	**Value**	**Parameter**	**Value**
penalty	l2	tol	0.0001	C	1.0	fit_intercept	True
dual	False	intercept_ scaling	1	class_weight	None	solver	Liblinear
max_iter	100	multi_class	Auto	verbose	0	random_ state	None
**KNN Parameters**							
**Parameter**	**Value**	**Parameter**	**Value**	**Parameter**	**Value**	**Parameter**	**Value**
n_neighbor	5	weight	Uniform	algorithm	Auto	leaf_ size	30
p	2	metric	Minkowski	metric_ param	None	n_jobs	None
**MNB Parameter**							
**Parameter**	**Value**	**Parameter**	**Value**	**Parameter**	**Value**	**Parameter**	**Value**
alpha	1.0	fit_prior	True	class_prior	None	alpha	1.0
**GNB Parameter**							
**Parameter**	**Value**	**Parameter**	**Value**	**Parameter**	**Value**	**Parameter**	**Value**
priors	None	var_smoothing	1 × 10^−9^	priors	None	var_smoothing	1 × 10^−9^
**DT Parameter**							
**Parameter**	**Value**	**Parameter**	**Value**	**Parameter**	**Value**	**Parameter**	**Value**
max_depth	8	max_features	None	max_leaf_node	None	min_sample_leaf	10
min_sample_split	2	random_state	11	splitter	Best		
